# Support usability, institutional trust, and psychological adaptation among international students from war-affected regions in Chinese universities: a mixed-methods study

**DOI:** 10.3389/fpsyg.2026.1913815

**Published:** 2026-08-06

**Authors:** Xujin Xian, Chenhe Zhao, Peitao Du

**Affiliations:** 1 The Catholic University of Korea, Bucheon, Republic of Korea; 2 Jining Polytechnic, Jining, Shandong, China; 3 Dongseo University, Busan, Republic of Korea; 4 Al-Farabi Kazakh National University, Almaty, Kazakhstan

**Keywords:** institutional trust, international students, mixed-methods study, organizational socialization, psychological adaptation, psychological safety, support usability, war-affected regions

## Abstract

Conflict-driven mobility is reshaping international higher education and raising new questions about how universities support students whose learning trajectories have been disrupted by war, political violence, or prolonged instability. Although international student adaptation has been widely examined through the lenses of cross-cultural adjustment, academic integration, and social support, less is known about how students from war-affected regions psychologically experience formal university support as visible, usable, trustworthy, and safe. This gap matters because support services may formally exist yet remain unused when students fear stigma, unclear procedures, confidentiality risks, or adverse institutional consequences. Here, we examined support usability, institutional trust, and psychological adaptation among international students from war-affected regions in Chinese universities through an exploratory sequential mixed-methods design. The qualitative phase included semi-structured interviews with 28 international students and 12 university staff members across five universities, together with analysis of 15 institutional documents. The second phase used the analytic hierarchy process (AHP) with 15 experts to evaluate the relative priority of support factors derived from the qualitative findings rather than to test causal relationships among student-level variables. Organizational support received the highest criterion-layer priority weight (0.382), followed by institutional trust (0.275), organizational socialization (0.223), and psychological safety (0.120). At the indicator level, timeliness of response ranked first (0.124), followed by perceived institutional trust (0.117), fairness of management systems (0.102), and availability of ongoing guidance (0.102). Qualitative findings further suggested that institutional trust may help explain formal help-seeking reluctance, organizational socialization can be interpreted as a continuous reparative process, and co-national peer networks may provide emotional shelter while also amplifying anxiety. Taken together, the qualitative findings and expert-derived priority rankings suggest that psychological adaptation among students from war-affected regions may be supported not only by the formal availability of university services but also by whether support is psychologically experienced as recognizable, approachable, trustworthy, and safe to use. By shifting attention from support provision to support usability, this study extends perceived organizational support theory and offers evidence-informed implications for designing trauma-sensitive, student-centered support systems in international higher education.

## 1 Introduction

Armed conflict increasingly disrupts not only lives and communities but also educational trajectories (Davies et al., 2024). As organized violence, political instability, and humanitarian crises continue to reshape global mobility patterns, a growing number of students from war-affected regions seek higher education in relatively stable host countries, including China (Davies et al., 2024; Cao and Meng, 2022). For these students, entering a university abroad is not simply a process of adjusting to a new language, curriculum, or culture. It may also involve managing traumatic memories, uncertainty about family safety, financial disruption, interrupted schooling, and weakened trust in formal institutions (Mazzero, 2025; Carpiniello, 2023). Their psychological adaptation therefore represents an important issue at the intersection of psychology, international higher education, trauma-informed support, and institutional responsibility.

International student adaptation is commonly examined through academic, sociocultural, and psychological dimensions (Cao and Meng, 2022; Soheili and Lanz, 2025). Previous studies have shown that language proficiency, cultural distance, academic adjustment, social support, friendship networks, and mental health services are closely related to international students’ well-being and educational outcomes (Cao and Meng, 2022; Sheng et al., 2022; Maharaj et al., 2025). However, students from war-affected regions may experience adaptation as a more complex process of rebuilding safety, predictability, belonging, help-seeking confidence, and educational continuity (Mazzero, 2025; Soufi Amlashi et al., 2024). Evidence from refugee and conflict-exposed populations indicates elevated risks of anxiety, depression, post-traumatic stress, and avoidance of formal help-seeking, especially when uncertainty and mistrust persist after migration (Blackmore et al., 2020; Mesa-Vieira et al., 2022; Byrow et al., 2020). Trauma-informed education further suggests that support systems must be experienced as safe, transparent, trustworthy, collaborative, and empowering before vulnerable students are likely to use them (Berring et al., 2024; Sakız and Jencius, 2024; Wu et al., 2021). In the Chinese university context, international students interact with multiple institutional units, including schools of international education, academic departments, counselors, supervisors, psychological counseling services, and administrative offices responsible for student status, scholarships, visas, and residence procedures (Cao and Meng, 2022; Wu et al., 2021). These systems can provide stability and support, but they may also appear complex, unfamiliar, or risky if procedures are unclear or if students fear that disclosure of distress may affect academic or administrative outcomes (Sakız and Jencius, 2024; Liu et al., 2024; Prado et al., 2024).

Although international student adaptation has been widely studied, several gaps remain. First, universities are often treated as background settings rather than as receiving organizations whose routines, support systems, and response practices shape students’ psychological experience of adaptation (Eisenberger et al., 2020; Bauer et al., 2025). Second, existing research has focused more on whether support resources exist than on whether students perceive such support as visible, approachable, trustworthy, and safe to use (Corney et al., 2024; Hardy et al., 2025; Akiba et al., 2024). This distinction is particularly important for students from war-affected regions, because formal services may remain unused when students worry about stigma, confidentiality, records, or institutional consequences (Byrow et al., 2020; Clement et al., 2015). Third, prior studies have identified multiple factors related to adaptation but have rarely clarified which support factors should be prioritized under real institutional resource constraints. To address these gaps, this study integrates perceived organizational support theory, organizational socialization theory, trauma-informed education, and forced-migration education perspectives. Perceived organizational support (POS) theory suggests that support becomes meaningful when individuals believe that the organization values their well-being and responds to their needs (Eisenberger et al., 2020). Organizational socialization theory explains how newcomers learn formal rules, implicit norms, and help-seeking pathways after entering an organization (Bauer et al., 2025; Woodrow and Guest, 2020). Applied to students from war-affected regions, these theories suggest that the key issue is not only support provision but also support usability: whether students can recognize, approach, trust, and safely use formal university support.

This study examines how support usability, perceived institutional trust, organizational socialization, and psychological safety are experienced in relation to the psychological adaptation of international students from war-affected regions in Chinese universities. Specifically, it asks how these students experience perceived organizational support, how organizational socialization is described as relevant to psychological adaptation and broader educational adjustment, and which support factors should be prioritized so that formal university systems are experienced as usable, trustworthy, and psychologically safe. To answer these questions, the study adopts an exploratory sequential mixed-methods design (Fetters et al., 2013; Guetterman et al., 2015). Semi-structured interviews with international students and university staff, together with institutional document analysis, are first used to identify core experiences and support factors. The analytic hierarchy process (AHP) is then used to evaluate the relative priority of qualitatively derived indicators for student-centered support design (Saaty, 2008; Emrouznejad and Marra, 2017; Subramanian and Ramanathan, 2012). By combining qualitative depth with structured priority assessment, this study aims to shift attention from the formal availability of support to its psychological usability, thereby contributing to research on international student adaptation, trauma-informed higher education, and organizational support for vulnerable student groups.

## 2 Theoretical background and framework

Because this study examines support usability, institutional trust, and psychological adaptation, the literature review is organized to move from general international student adaptation to the distinctive vulnerabilities of students from war-affected regions and then to the organizational mechanisms through which university support becomes psychologically usable. Existing research has separately examined cross-cultural adjustment, trauma-related vulnerability, social support, and institutional services, but it has less often explained how these domains combine to shape whether vulnerable students recognize, trust, and use formal support (Soufi Amlashi et al., 2024; Carpiniello, 2023). The following framework therefore treats psychological adaptation as a process shaped by both individual stressors and students’ psychological experience of the university as a receiving organization (Edmondson and Bransby, 2023).

### 2.1 Background

#### 2.1.1 International student adaptation

International student adaptation is commonly understood as a multidimensional process involving academic, psychological, and sociocultural adjustment (Cao and Meng, 2022; Soheili and Lanz, 2025). Academic adaptation refers to students’ ability to participate in courses, understand academic expectations, interact with teachers and peers, and maintain learning performance (Cao and Meng, 2022; Sheng et al., 2022). In this study, psychological adaptation refers not only to emotional stability, stress regulation, belonging, and subjective well-being, but also to students’ perceived safety, trust in institutional systems, willingness to seek help, and ability to rebuild predictability and action certainty in the university environment (Soufi Amlashi et al., 2024; Zhang et al., 2026). Academic and sociocultural adjustment are treated as closely related contextual domains that interact with psychological adaptation (Sheng et al., 2022).

For international students from war-affected regions, psychological adaptation is embedded in additional layers of vulnerability (Blackmore et al., 2020; Mesa-Vieira et al., 2022; Handiso et al., 2026). War-related experiences may include direct exposure to violence, family separation, loss of economic stability, disruption of prior schooling, and uncertainty about returning home (Davies et al., 2024; Carpiniello, 2023; Mazzero, 2025). Even after entering a safer educational environment, students may continue to worry about family members, home-country instability, financial pressures, and interrupted life plans (Mazzero, 2025; Nguyen et al., 2024). These pressures make their adaptation qualitatively different from ordinary cross-cultural adjustment.

Psychological adaptation in this group should therefore be understood not only as emotional adjustment to a new language or culture, but also as the rebuilding of felt safety, educational continuity, help-seeking confidence, and confidence in formal institutions (Byrow et al., 2020; Sakız and Jencius, 2024; Wu et al., 2021). This requires a framework that connects international student adaptation research with trauma-informed education and forced-migration education while also explaining how university support systems become psychologically recognizable, approachable, and trustworthy enough to use (Berring et al., 2024).

#### 2.1.2 The distinctiveness of students from war-affected regions

International students from war-affected regions are not simply an intensified version of ordinary international students. Their adaptation is shaped by intersecting psychological, educational, and institutional pressures (Davies et al., 2024; Carpiniello, 2023; Mazzero, 2025). First, traumatic memories and exposure to insecurity may increase anxiety, avoidance, and sensitivity to uncertainty (Blackmore et al., 2020; Henkelmann et al., 2020; Mesa-Vieira et al., 2022). Second, continuing anxiety about family safety may make emotional regulation difficult, even when students are physically distant from conflict. Third, economic disruption and family instability may reduce financial and emotional support. Fourth, uncertainty about home-country conditions may affect students’ future planning, study motivation, and sense of control (Mazzero, 2025).

These characteristics suggest that support for this group should not be understood only as the provision of language training, cultural activities, or general international student services. From the students’ perspective, formal support must be experienced as predictable, confidential, low-risk, and psychologically safe before it can become actionable (Byrow et al., 2020; Corney et al., 2024; Hardy et al., 2025). Students may also need sustained guidance to understand how university systems operate and whether formal help-seeking will have any negative consequences for academic evaluation, scholarships, visas, or residence procedures (Clement et al., 2015; Byrow et al., 2020). Thus, the central issue is not only whether support exists, but whether students experience it as safe and usable in practice.

#### 2.1.3 Trauma-informed and forced-migration education perspectives

A trauma-informed education perspective emphasizes safety, trustworthiness, transparency, peer support, collaboration, empowerment, and sensitivity to cultural and historical experiences (Berring et al., 2024; Alessi and Kahn, 2023; Grailey et al., 2021). These principles are important because students affected by war-related instability may interpret unclear procedures, delayed responses, or ambiguous institutional messages as threatening (Alessi and Kahn, 2023; Berring et al., 2024). Support systems therefore cannot be evaluated only by whether resources exist. They must also be evaluated by whether students experience them as safe to approach and reliable to use (Edmondson and Bransby, 2023).

Forced-migration education research emphasizes the repair of educational continuity after displacement, disruption, or prolonged uncertainty (Mazzero, 2025; Dryden-Peterson et al., 2017; Nguyen et al., 2024). In higher education, this repair is not only administrative but also psychological: students need to experience learning routines as stable, institutional expectations as interpretable, support as approachable, and future planning as possible again (Mazzero, 2025; Dryden-Peterson et al., 2017). Trauma-informed and forced-migration perspectives therefore explain why safety, trust, transparency, empowerment, and continuity matter. However, they do not fully specify how university organizations translate formal services into support that students perceive as available and safe to use. Perceived organizational support theory and organizational socialization theory address this next step by explaining how organizational responsiveness, reliable relationships, and continuous rule-learning pathways can make safety and continuity psychologically accessible to war-affected students (Eisenberger et al., 2020; Bauer et al., 2025).

### 2.2 Theoretical framework

#### 2.2.1 Perceived organizational support and organizational socialization

Perceived organizational support theory argues that support becomes meaningful when members believe that the organization values their well-being and responds to their needs (Eisenberger et al., 2020; Kurtessis et al., 2017). In the context of international student education, the university can be understood as a receiving organization whose formal support includes counseling, academic guidance, financial assistance, visa-related services, student-status support, and emergency assistance (Wu et al., 2021; Sakız and Jencius, 2024). For the present study, perceived organizational support is not treated as support quantity alone. It is treated as a psychological perception: support becomes relevant to adaptation only when students experience the organization as visible, accessible, responsive, reliable, and safe to approach (Eisenberger et al., 2020; Corney et al., 2024).

Building on this distinction, support usability is defined in this study as the extent to which formal support is psychologically experienced by students as recognizable, approachable, and trustworthy. It is therefore not only a service-management feature, but a psychological condition through which institutional support becomes subjectively available and actionable (Sakız and Jencius, 2024; Corney et al., 2024). Recognizability means that students can identify what support exists and where to find it. Approachability means that procedures are simple, low-threshold, and culturally understandable. Trustworthiness means that students believe using support will not create stigma, records, or institutional risk (Byrow et al., 2020; Hardy et al., 2025; Clement et al., 2015). This three-layer structure clarifies why support provision does not automatically become perceived organizational support: formal support matters not merely because it exists, but because students psychologically experience it as recognizable, approachable, and trustworthy enough to use.

Organizational socialization theory explains how newcomers learn explicit rules, implicit norms, expected behaviors, and help-seeking pathways after entering an organization (Bauer et al., 2025; Woodrow and Guest, 2020). For international students, entering a university is both a cross-cultural transition and an organizational entry process (Cao and Meng, 2022; Cao et al., 2024). They must learn how teaching systems, administrative procedures, academic expectations, campus norms, and student services operate. This process cannot be completed through one-off orientation alone (Bauer et al., 2025; Woodrow and Guest, 2020). For students whose prior educational continuity has been disrupted, socialization may also involve rebuilding confidence in predictable rules and reliable institutional relationships (Mazzero, 2025). In this sense, organizational socialization functions as a psychological mechanism through which students gradually regain interpretability, predictability, and action certainty in the receiving university environment.

In this study, perceived institutional trust refers to students’ judgment that university systems are reliable, fair, confidential, and safe to use when help is needed. It differs from institutional transparency, which concerns whether information is open and understandable, and from fairness of management systems, which concerns the justice of procedures and outcomes. It is also narrower than general organizational trust because it focuses specifically on whether formal university systems can be approached without stigma, record-keeping risks, or adverse institutional consequences (Byrow et al., 2020; Clement et al., 2015; Hardy et al., 2025). Perceived institutional trust is therefore conceptualized as an activation condition for formal help-seeking. Put simply, psychological safety concerns whether students feel able to speak up and seek help in a specific situation, whereas perceived institutional trust concerns whether they believe the university system is reliably fair, confidential, and non-punitive over time (Edmondson and Bransby, 2023; Grailey et al., 2021).

#### 2.2.2 Integrated theoretical framework

The theoretical framework is built from the literature gap identified above: existing research has explained many aspects of international student adjustment, trauma-related vulnerability, social support, and institutional service provision, but it has less often specified how formal support becomes psychologically actionable for war-affected international students (Cao and Meng, 2022; Soufi Amlashi et al., 2024; Mazzero, 2025). The framework therefore treats psychological adaptation as embedded in broader academic, organizational, and sociocultural adjustment. It focuses on how students psychologically experience the university as a receiving organization while trying to rebuild learning stability, institutional confidence, and psychological safety. War-related cumulative stressors, including traumatic memories, family safety anxiety, economic disruption, and interrupted learning continuity, create heightened adaptation needs (Carpiniello, 2023; Mesa-Vieira et al., 2022; Nguyen et al., 2024). Organizational mechanisms, especially perceived organizational support and organizational socialization, are conceptualized as conditions that may be associated with whether students experience formal systems as predictable, interpretable, and safe to use (Eisenberger et al., 2020; Bauer et al., 2025). Perceived institutional trust, psychological safety, and co-national peer networks are treated as activation and contextual conditions that may be relevant to whether formal support is experienced as usable and whether organizational socialization can proceed effectively (Byrow et al., 2020; Edmondson and Bransby, 2023; Bender et al., 2019).

**Figure 1:**
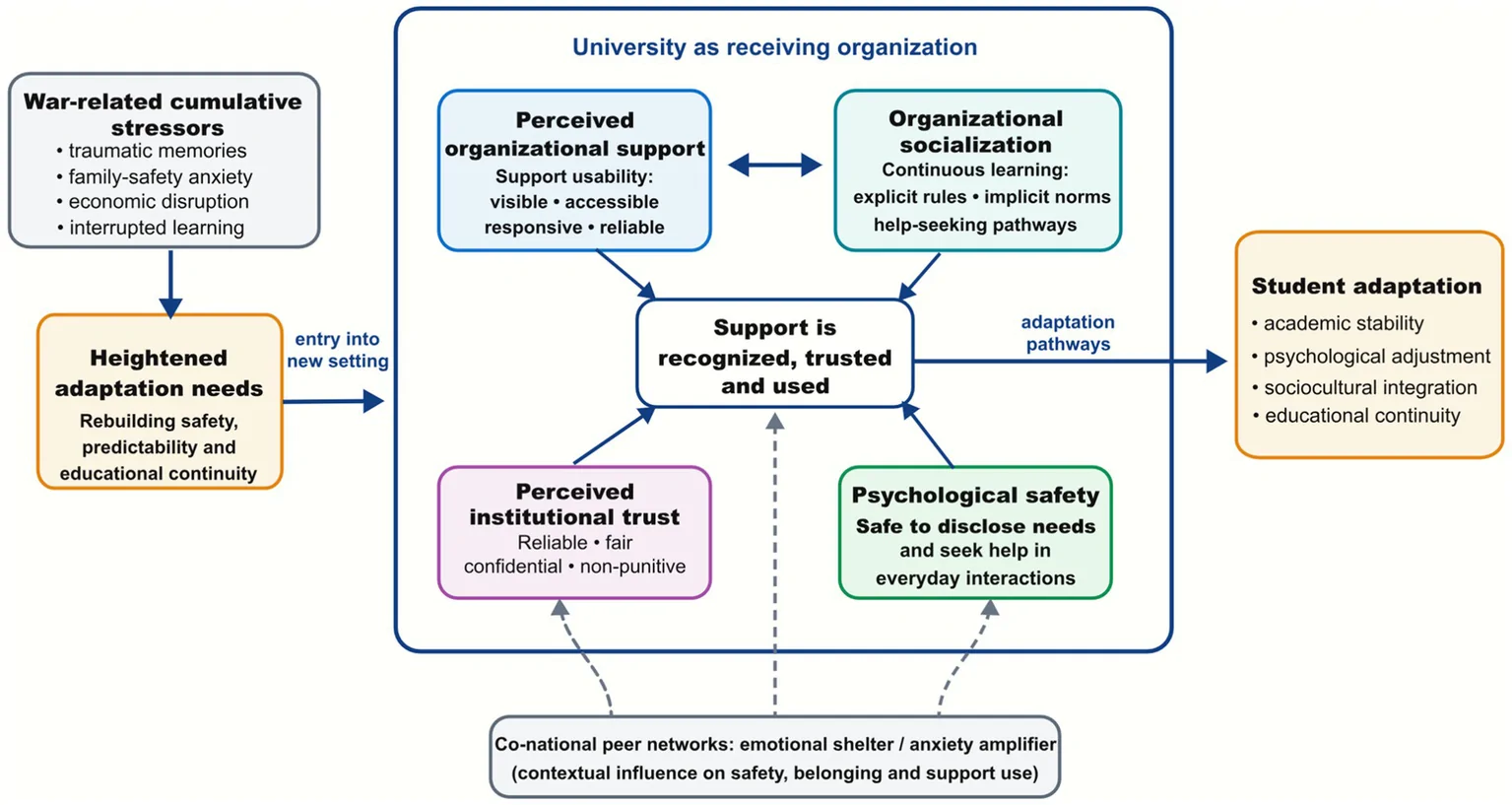
Theoretical framework of the study. The framework conceptualizes the university as a receiving organization. War-related cumulative stressors are conceptualized as conditions associated with heightened adaptation needs, while perceived organizational support and organizational socialization are treated as central organizational mechanisms. Perceived institutional trust, psychological safety, and co-national peer networks are conceptualized as activation and contextual conditions that may influence whether formal support is experienced as usable and whether organizational socialization can proceed effectively.

In this framework, perceived institutional trust and psychological safety are related but conceptually distinct. Perceived institutional trust refers to students’ evaluation of formal university systems as reliable, fair, confidential, and non-punitive. Psychological safety refers to students’ felt safety in expressing needs, disclosing difficulties, and seeking help in everyday campus interactions (Edmondson and Bransby, 2023; Grailey et al., 2021). Institutional trust can therefore be understood as a system-level condition that supports psychological safety, while low psychological safety may prevent students from acting on available support even when formal services exist (Byrow et al., 2020; Clement et al., 2015). This distinction is important for the full mixed-methods design because the qualitative phase examines how students interpret these conditions, whereas the AHP phase prioritizes support factors that may make formal systems more usable, trustworthy, and safe under resource constraints (Figure 1).

The AHP is used in this study because prior research has identified many adaptation factors but rarely assessed their relative priority (Saaty, 2008; Emrouznejad and Marra, 2017; Subramanian and Ramanathan, 2012). Under resource constraints, it is important to know which support factors are most likely to make formal systems psychologically usable, trustworthy, and safe for students. AHP translates qualitatively identified factors into a structured hierarchy and uses expert judgments to determine relative weights (Saaty, 2008). In this study, AHP functions as a decision-support tool for interpreting support priorities in relation to students’ psychological adaptation and broader adjustment rather than as a statistical causal test (Saaty, 2008; Krejci and Stoklasa, 2018). This placement also aligns the theoretical framework with the empirical design: qualitative findings explain students’ lived psychological experience of support, while AHP clarifies which support conditions should be prioritized to improve support usability.

## 3 Materials and methods

### 3.1 Research design

This study adopts an exploratory sequential mixed-methods design (Fetters et al., 2013; Guetterman et al., 2015). The qualitative-first, AHP-second path is appropriate because no mature indicator system yet exists for the psychological adaptation and broader educational adjustment of international students from war-affected regions in Chinese universities. The qualitative phase identifies core experiences and candidate support factors, while the AHP phase ranks these factors as expert-derived support-design priorities for psychologically usable, trustworthy, and safe help-seeking pathways under resource constraints (Saaty, 2008).

The two phases serve different purposes. The qualitative phase explains how students and staff experience support, socialization, trust, safety, and peer networks in relation to psychological adaptation. The AHP phase determines expert-based priorities for support usability rather than testing statistical causality (Saaty, 2008). Integration occurs after the AHP phase by aligning qualitative themes with corresponding AHP indicators and global weights in a joint display table (Guetterman et al., 2015). This design allows the study to combine explanatory depth with structured expert priority assessment (Fetters et al., 2013; Guetterman et al., 2015).

The mixed-methods contribution lies in the sequential transformation of evidence across phases. Qualitative interview and document-analysis codes were first organized into subthemes and core themes, then translated into criterion-layer dimensions and actionable AHP indicators. The AHP phase subsequently ranked these qualitatively derived indicators, and the final interpretation integrated both phases by using interview evidence to explain why highly ranked indicators were meaningful for students’ support-use experiences. This transformation pathway is summarized in the joint display and in Supplementary Table S12.

#### 3.1.1 Phase 1: qualitative research

The first phase combined semi-structured interviews and document analysis to identify psychological adaptation experiences and candidate support factors (Palinkas et al., 2015; Dalglish et al., 2020; Kiger and Varpio, 2020). Five Chinese universities were selected, including a comprehensive key university, a science and engineering university, a language university, a finance and economics university, and a teacher education university. These universities differed in institutional type, support structure, and international student management practices, which provided comparative empirical material.

The sample was obtained through purposive and snowball sampling (Palinkas et al., 2015). It included 28 international students from war-affected regions and 12 university staff members, including teachers from schools of international education, counselors, psychological counseling teachers, and administrative personnel. Staff participants were included only if they had direct or closely related experience supporting international students from war-affected regions or other high-vulnerability contexts, such as cases involving disrupted schooling, family-safety concerns, visa or student-status uncertainty, or crisis-related adaptation. For sampling purposes, war-affected regions referred to home countries or areas that had recently experienced, or were continuing to experience, armed conflict, political violence, humanitarian crisis, or prolonged instability according to public conflict and humanitarian information (Davies et al., 2024; Carpiniello, 2023). This contextual label was used to guide recruitment and protect confidentiality; it was not treated as proof of individual traumatic exposure, refugee status, or a homogeneous conflict experience (Alessi and Kahn, 2023). To improve methodological transparency while protecting confidentiality, the student sample was summarized at a non-identifying regional level rather than at the country or nationality level. Participants came from the Middle East, North Africa, Sub-Saharan Africa, and other conflict-affected regions. Because several possible combinations of country or area, degree level, gender, university type, and language background involved very small cells, exact country names, nationalities, religions, and small-cell counts are not reported. Detailed anonymized regional and demographic distributions are provided in Supplementary Tables S1, S2 and Table 1.

**Table 1:** Overview of interviewees and expert samples.

Sample type	Number	Role	Source
International students	28	Undergraduate, master’s, and doctoral students	From non-identifying conflict-affected regional categories: Middle East, North Africa, Sub-Saharan Africa, and other conflict-affected regions. Exact country, nationality, and small-cell counts are not reported to prevent deductive identification. See Supplementary Tables S1, S2 for anonymized distributions.
University staff	12	Teachers from schools of international education, counselors, psychological counseling teachers, and administrative personnel	From the five sample universities; all had more than 3 years of experience in the management and support of international students, including direct or closely related experience with students from war-affected regions or other high-vulnerability international student cases.
AHP experts	15	Experts in international education research (5), university international student management experts (5), psychology experts (5)	From universities and research institutions; all had more than 5 years of relevant research or work experience

To protect confidentiality, participants’ specific nationalities, countries, areas of origin, universities, religions, and language backgrounds are not reported (Alessi and Kahn, 2023; World Medical Association, 2025). Instead, broad regional descriptions and anonymized participant codes are used where necessary. Public situation reports and conflict-event sources were consulted only as contextual references; no personal data were cross-referenced with external databases (Davies et al., 2024). This reporting strategy preserves analytic transparency at the level of broad conflict-affected contexts while reducing the risk of deductive disclosure in a small and vulnerable participant group (Alessi and Kahn, 2023). It also means that the study does not compare outcomes across specific countries, nationalities, or conflict types.

Semi-structured interviews were the core data source (Alessi and Kahn, 2023). Each interview lasted approximately 40–60 min. Chinese or English was used according to participant preference. Interviews were audio-recorded with consent and transcribed. Interviews conducted in English were transcribed verbatim and translated into Chinese by bilingual members of the research team. Translations were checked against the original transcripts, and discrepancies were discussed until semantic agreement was reached. Key terms, including support usability, institutional trust, psychological safety, organizational socialization, and adaptation, were standardized before coding to reduce translation-related bias (Regmi et al., 2010). The semi-structured interview guides for international students and university staff are provided in Supplementary Material S3.

The interview guides were developed from perceived organizational support theory, organizational socialization theory, trauma-informed education, and forced-migration education literature. Questions were reviewed for trauma-sensitive wording and were designed to focus on campus adaptation and support-use experiences rather than requiring participants to recount traumatic events directly. The guide was also checked for clarity and conceptual coverage before formal interviews.

Document analysis supplemented and cross-validated the interview data (Dalglish et al., 2020). Fifteen documents were reviewed, including international student regulations, student handbooks, visa and student-status procedures, psychological support descriptions, annual work reports, and student notices. Document analysis helped clarify institutional design, information visibility, and formal support procedures (Dalglish et al., 2020). The documents included in the analysis are listed in Supplementary Table S4.

Sample size was determined by information saturation and information power (Malterud et al., 2016; Saunders et al., 2018). After the 24th student interview and the 10th staff interview, new themes decreased markedly. Additional interviews confirmed theme stability. The study therefore prioritized thematic sufficiency and explanatory depth rather than statistical representativeness (Malterud et al., 2016).

#### 3.1.2 Qualitative data analysis

The study used thematic analysis informed by grounded theory coding logic (Braun and Clarke, 2021; Kiger and Varpio, 2020). The research team read transcripts and documents paragraph by paragraph, conducted initial coding close to participants’ expressions, and marked content related to organizational support, organizational socialization, perceived institutional trust, psychological safety, and co-national peer networks. Similar codes were then clustered into subthemes and integrated into explanatory core themes through cross-material comparison (Kiger and Varpio, 2020).

The analysis generated 157 initial codes, which were merged into 12 subthemes and 4 core themes. Two researchers independently completed the first coding round and cross-checked results (Nowell et al., 2017). Disagreements were resolved by returning to original texts, discussing conceptual boundaries, and comparing typical excerpts. Although the analysis drew on the inductive logic of grounded theory coding, it is better described as thematic analysis informed by grounded theory because the study aimed to identify experiential themes within an existing theoretical framework rather than to build a completely new process theory (Braun and Clarke, 2021; Kiger and Varpio, 2020). Detailed qualitative themes, subthemes, and coding examples are provided in Supplementary Table S5.

#### 3.1.3 Trustworthiness and rigor

Trustworthiness was strengthened through credibility, transferability, dependability, and confirmability (Nowell et al., 2017; Braun and Clarke, 2021). Credibility was supported by independent coding, researcher comparison, and limited trauma-sensitive member checking (Alessi and Kahn, 2023). Two researchers coded a subset of transcripts independently, compared results, and developed the final framework. Intercoder reliability showed substantial agreement, with Cohen’s kappa = 0.81. Limited member checking returned summarized themes to willing participants without requiring them to revisit distressing details or confirm sensitive quotations (Alessi and Kahn, 2023). Additional inter-coder reliability details are provided in Supplementary Table S6.

Transferability was supported by reporting participant roles, institutional types, and broad war-affected contexts while preserving anonymity (Nowell et al., 2017; Alessi and Kahn, 2023). Dependability was supported by retaining interview protocols, coding memos, theme-development notes, and records of transforming qualitative themes into AHP indicators. Confirmability was strengthened through team reflexive discussions about how researchers’ disciplinary backgrounds and assumptions about vulnerability might shape interpretation (Nowell et al., 2017). The research team included members with backgrounds in education, psychology, and international student support. During analysis, the team explicitly discussed how assumptions about trauma, institutional responsibility, and student vulnerability could influence coding decisions, theme naming, and the interpretation of support needs.

### 3.2 Phase 2: AHP study

#### 3.2.1 Rationale for using AHP instead of structural equation modeling (SEM) or regression

The AHP phase aimed to generate a structured priority ranking for student-centered support design rather than to test causal relationships (Saaty, 2008; Emrouznejad and Marra, 2017). Structural equation modeling (SEM) and regression address associations among empirically measured variables, whereas AHP addresses the relative priorities assigned to decision criteria by a structured expert panel (Saaty, 2008). AHP was therefore selected because the second phase sought to rank support factors according to their importance for psychologically usable and trustworthy help-seeking pathways, which are interpreted here as conditions relevant to psychological adaptation, rather than estimate associations or causal effects. The small and difficult-to-sample target population and the absence of mature validated instruments further supported the qualitative-first design, but AHP was not treated as a small-sample substitute for SEM or regression (Subramanian and Ramanathan, 2012; Emrouznejad and Marra, 2017).

The AHP findings should therefore be read as aggregated expert judgments about support-design priority, not as causal effect sizes (Saaty, 2008). In this mixed-methods design, qualitative findings explain lived experiences and interpretive processes, while AHP clarifies which factors should be prioritized to improve the psychological usability and trustworthiness of formal support under institutional resource constraints (Fetters et al., 2013; Guetterman et al., 2015; Saaty, 2008).

#### 3.2.2 Construction of the AHP indicator system

The AHP indicator system was constructed from the qualitative themes rather than imposed as a separate quantitative model (Saaty, 2008). Interview and document-analysis codes were first grouped into subthemes, then condensed into four criterion-layer dimensions and 12 actionable indicators. The target layer was ‘priority of key support factors for psychological adaptation and related adjustment among international students from war-affected regions.’ The criterion layer contained four dimensions: organizational support, institutional trust dimension, organizational socialization, and psychological safety. Although the AHP questionnaire used the shorter wording ‘adaptation’ for readability, the resulting priorities are interpreted in this article in relation to students’ psychological adaptation and related academic and sociocultural adjustment. Each criterion-layer dimension was then specified into concrete indicators, forming a three-level system of target, criterion, and indicator layers (Saaty, 2008; Eisenberger et al., 2020; Bauer et al., 2025). The transformation from qualitative findings to AHP indicators is documented in Supplementary Table S12.

Organizational support included availability of support information, simplicity of help-seeking procedures, timeliness of response, and adequacy of support resources (Eisenberger et al., 2020; Sakız and Jencius, 2024; Corney et al., 2024). The institutional trust dimension included perceived institutional trust, fairness of management systems, and institutional transparency (Byrow et al., 2020; Clement et al., 2015). Organizational socialization included availability of ongoing guidance, channels for learning implicit rules, and awareness of organizational rules (Bauer et al., 2025). Psychological safety included campus safety climate and support for the establishment of belonging (Edmondson and Bransby, 2023). Table 2 presents the resulting three-level AHP indicator system.

**Table 2:** AHP indicator system for support priorities.

Target layer	Criterion layer	Indicator layer
Priority of key support factors for psychological adaptation and related adjustment among international students from war-affected regions	Organizational support	Availability of support information
		Simplicity of help-seeking procedures
		Timeliness of response
		Adequacy of support resources
	Institutional trust dimension	Perceived institutional trust
		Fairness of management systems
		Institutional transparency
	Organizational socialization	Availability of ongoing guidance
		Channels for learning implicit rules
		Awareness of organizational rules
	Psychological safety	Campus safety climate
		Support for the establishment of belonging

The psychological safety dimension contains two directly actionable indicators: campus safety climate and support for the establishment of belonging. Co-national peer networks are discussed qualitatively rather than as an independent AHP criterion because the AHP system prioritizes formal support conditions that can be intentionally designed to shape students’ experience of safety, belonging, and trust.

Although co-national peer networks emerged as a core qualitative theme, they were not included as an independent AHP criterion (Bender et al., 2019; Sheng et al., 2022; Nguyen et al., 2024). The AHP system prioritized formal conditions through which support can be made psychologically usable, including socialization, trust-building, and safety mechanisms (Saaty, 2008; Corney et al., 2024; Edmondson and Bransby, 2023). Co-national peer networks are therefore discussed qualitatively and linked to psychological safety and belonging. This improves actionability but may underrepresent informal peer processes (Bender et al., 2019; Nguyen et al., 2024).

#### 3.2.3 Expert selection and questionnaire design

Fifteen experts participated in the AHP evaluation (Saaty, 2008). The panel included 5 experts in international education research, 5 experts in university international student management, and 5 experts in psychology. All had more than 5 years of relevant research or work experience. This panel size was selected to balance disciplinary breadth, AHP feasibility, and the need for informed judgments from fields directly related to international student support and trauma-sensitive adaptation. The purpose was not to obtain statistically representative expert opinions, but to aggregate structured judgments from three relevant professional perspectives. In addition to disciplinary background, experts were invited only if they reported prior experience with international student support, cross-cultural adaptation, vulnerable student groups, displacement-related education, trauma-informed support, or related counseling and management issues. Education researchers had published relevant academic work, management experts had direct experience in international student management, and psychology experts had experience in trauma psychology, counseling, or cross-cultural intervention. The profiles of participating AHP experts are summarized in Supplementary Table S7.

The AHP questionnaire used pairwise comparisons at criterion and indicator layers with the 1–9 scale method, where 1 indicates equal importance and 9 indicates extreme importance of one factor over another (Saaty, 2008; Emrouznejad and Marra, 2017). Before formal administration, the AHP questionnaire was pilot-tested with three scholars familiar with international student support and mixed-methods research to ensure that the wording of the indicators and pairwise comparison tasks was clear and conceptually appropriate. Minor revisions were made to improve clarity and reduce ambiguity in several indicator labels. The formal expert panel subsequently completed the revised questionnaire independently. Fifteen valid questionnaires were collected, yielding a valid response rate of 100%. The complete AHP expert rating questionnaire, including all pairwise-comparison items, is provided in Supplementary Material S8.

#### 3.2.4 Weight calculation and consistency test

Weight calculation followed standard AHP procedures (Saaty, 2008; Emrouznejad and Marra, 2017). First, individual questionnaires were checked for completeness before the expert judgments were aggregated. Group judgment matrices were then constructed by aggregating experts’ ratings using the geometric mean, which is widely recommended for group AHP because it preserves the reciprocal properties of pairwise comparisons (Krejci and Stoklasa, 2018). Maximum eigenvalues and corresponding eigenvectors were calculated for each group matrix, and normalized eigenvectors were used to derive local weights at the criterion and indicator levels (Saaty, 2008). Global weights were obtained by multiplying the local weight of each indicator by the weight of its corresponding criterion-layer dimension. At the reporting stage, the study emphasizes aggregated weights, consistency statistics, and questionnaire transparency; individual expert matrices are not reported because they could reveal potentially identifying response patterns in a small expert panel.

Consistency was evaluated using the consistency index (CI) and consistency ratio (CR), with CR < 0.10 indicating acceptable consistency (Saaty, 2008). All aggregated judgment matrices for which CR was applicable satisfied this criterion, indicating acceptable internal consistency of the aggregated pairwise comparisons (Emrouznejad and Marra, 2017). Table 3 reports the consistency results. The aggregated criterion-layer pairwise comparison judgment matrix is provided in Supplementary Table S9.

**Table 3:** Consistency test results of the AHP judgment matrices.

Judgment matrix	*n*	Lambda max	CI	RI	CR	Consistency assessment
Criterion	4	4.072	0.024	0.89	0.027	Acceptable
Org. support	4	4.09	0.03	0.89	0.034	Acceptable
Inst. trust	3	3.018	0.009	0.52	0.017	Acceptable
Org. socialization	3	3.025	0.013	0.52	0.024	Acceptable
Psych. safety	2	2	0.00	0.00	N/A	Not required for a reciprocal 2 × 2 matrix

CI = (lambda max – *n*)/(*n* – 1), and CR = CI/RI (Saaty, 2008). For matrices with *n* ≥ 3, CR < 0.10 indicates acceptable internal consistency. For the two-item psychological safety matrix, CR is not applicable because any reciprocal 2 × 2 comparison matrix is perfectly consistent.

Because each disciplinary subgroup contained only five experts, subgroup-specific AHP weights were not treated as separate inferential evidence. The equal composition of the panel was used to reduce the risk that the final priorities would be dominated by a single disciplinary perspective. The expert panel was intended to provide structured decision priorities rather than population-level generalizable estimates (Saaty, 2008; Guetterman et al., 2015). Nevertheless, future studies with larger expert panels should report subgroup weight comparisons among international education researchers, student management experts, and psychology experts to test the robustness of priority rankings across professional perspectives.

### 3.3 Integration analysis

Integration aligned qualitative themes, corresponding AHP indicators, and global weights in a joint display table (Guetterman et al., 2015; Fetters et al., 2013). The analysis examined convergence and divergence between interview-based interpretations and expert-derived priority rankings. Qualitative findings were used to explain why highly ranked support conditions were psychologically meaningful for students, whereas AHP weights were interpreted as priority rankings for support design rather than causal evidence or student-level effect sizes. This integration produced conclusions that combined qualitative depth with structured expert priority assessment (Fetters et al., 2013; Guetterman et al., 2015).

### 3.4 Ethical statement

This study was conducted in accordance with the Declaration of Helsinki (World Medical Association, 2025) and was reviewed and approved by the institutional ethics review body of Asia-Pacific Think Tank Research Institute under Approval No. APTTRI-IRB-2026-384729 on January 5, 2026. The ethics review confirmed that the study did not involve live animal experiments, clinical trials, biomedical intervention, or the collection of human biological specimens. Before participation, all participants were informed of the research purpose, procedures, data use, privacy protection measures, and their right to withdraw at any time (World Medical Association, 2025). Written informed consent was obtained from all participants. The informed consent form is provided in Supplementary Material S10.

Because some participants came from war-affected regions and might have traumatic experiences, the research team adopted trauma-sensitive interviewing methods to reduce the risk of triggering traumatic recollections (Alessi and Kahn, 2023; Berring et al., 2024). Participants were allowed to pause, skip questions, or withdraw from the interview at any time, and referral information for university support services was available if emotional distress occurred (Alessi and Kahn, 2023). For example, instead of asking directly about exposure to war violence, interviewers asked broader and less intrusive questions about students’ living and learning environments before coming to China. All personal identifiers were removed, and all data were securely stored and used only for this study (Alessi and Kahn, 2023; World Medical Association, 2025).

## 4 Results

### 4.1 Qualitative findings

Thematic analysis of interviews with 28 international students from war-affected regions and 12 university staff members identified four core themes relevant to psychological adaptation: the perception mechanism of organizational support, the continuous nature of organizational socialization, the dual role of co-national peer networks, and the influence of perceived institutional trust and psychological safety. Table 4 summarizes the core meanings and representative quotations.

**Table 4:** Qualitative themes and representative quotations.

Theme	Core meaning	Representative quotation (example)
Theme 1: Support must be visible, accessible, and responsive to be perceived as real	Students’ perception of university organizational support does not center on the amount of resources, but on whether support information is visible, help-seeking channels are accessible, and timely responses are received after help is sought.	“The university said there was psychological counseling, but I could not find the application procedure, and when I asked the counselor, there was no clear reply.” (S08)
Theme 2: Organizational socialization is a continuous learning process	International students’ organizational socialization is not completed by one-off orientation, but occurs through everyday interaction, repeated explanation, trial and error, and relational support.	“Only by talking with senior students and trying multiple times did I gradually learn the methods.” (S12)
Theme 3: Co-national peer networks are both emotional shelter and anxiety amplifier	Co-national peer networks can provide companionship, information, and familiar language, but they may also transmit anxiety and limit wider campus integration.	“Sometimes after talking with people from my own region, I become even more anxious, because everyone is worried about things back home.” (S21)
Theme 4: Institutional mistrust and low psychological safety can make formal support dormant	Students’ distrust of university systems and fear of institutional consequences mean that existing resources may remain unused in practice.	“I worry that applying for psychological support will be recorded by the university and affect my visa renewal.” (S24)

#### 4.1.1 Support must be visible, accessible, and responsive to be perceived as real

Interviews showed that students did not evaluate university support primarily by the amount of available resources, but by whether support was visible, accessible, and responsive in practice. Several participants described situations in which support services formally existed but remained difficult to use because procedures were unclear, information was fragmented, or responses were delayed.

One master’s student from a broad Middle Eastern region (S08) noted: “The university said there was psychological counseling and academic help, but I could not find the specific application procedure, and when I asked the counselor, I did not get a clear reply. In the end, I gave up.” Another student (S17) explained, “When I do not understand a deadline or a form, I become very nervous. I do not know who is safe to ask.”

These accounts suggest that, for war-affected students, procedural ambiguity and delayed response may transform formally available support into functionally unusable support. In other words, support does not become psychologically meaningful simply because resources exist. Students must be able to locate support, understand how to access it, and receive a timely and reliable response.

#### 4.1.2 Organizational socialization is a continuous learning process

Organizational socialization was not experienced as a one-off orientation event, but as a continuous process of learning how the university actually operates. Orientation helped students understand basic rules, but participants emphasized that meaningful adaptation occurred gradually through repeated interaction, trial and error, and ongoing relational support.

One student (S12) commented, “Only by talking with senior students and trying multiple times did I gradually learn the methods.” This statement reflects the gap between formal orientation and everyday institutional learning. Students often needed time to understand academic expectations, administrative procedures, teacher-student interaction norms, scholarship rules, student-status management, and help-seeking channels.

Staff interviews also indicated that international students often required repeated explanations after the initial orientation period. This finding suggests that organizational socialization in this group is better understood as a sustained and reparative learning process rather than a discrete entry event. For students from war-affected regions, learning how the university operates can help rebuild predictability, interpretability, and action certainty.

#### 4.1.3 Co-national peer networks are both emotional shelter and anxiety amplifier

Co-national peer networks played a distinctly ambivalent role. On the one hand, they provided emotional companionship, practical information, shared language, and a sense of familiarity that reduced isolation. On the other hand, they sometimes intensified exposure to distressing news and anxiety about home-country conditions.

One participant (S21) stated, “Sometimes after talking with people from my own region, I become even more anxious, because everyone is worried about things back home.” This quotation shows that peer networks may transmit not only support but also anxiety. Some students also reported that spending most of their time within co-national circles made it harder to interact with Chinese students or the wider campus community.

These findings suggest that co-national peer networks should not be viewed as uniformly beneficial or harmful. They function as both emotional shelter and potential anxiety amplifier. Peer networks should therefore be understood as important informal psychological environments that can either support adaptation or reinforce emotional distress and social closure.

#### 4.1.4 Institutional mistrust and low psychological safety can make formal support dormant

Insufficient perceived institutional trust and psychological safety were major reasons why formal support systems remained dormant. Some students worried that help-seeking might mark them as “problem students,” affect academic evaluations, or be linked to scholarship, visa, or residence matters.

One doctoral student (S24) said: “I worry that applying for psychological support will be recorded by the university and affect my later visa renewal, so even when I am under great stress, I do not dare seek help.” Another participant (S06) added, “Back home, asking the wrong office could bring trouble. I know it is different here, but I still feel afraid.”

These accounts show that transparent rules alone are insufficient. Students must also experience university systems as confidential, fair, and psychologically safe before formal support can be approached and used. Without perceived institutional trust, psychological support, academic tutoring, and other services may exist institutionally but remain unused in practice.

### 4.2 AHP findings

Fifteen valid AHP questionnaires were collected. All aggregated judgment matrices for which CR was applicable had CR values below 0.10, indicating acceptable internal consistency of the aggregated pairwise comparisons.

The reported weights are therefore interpreted as aggregated cross-disciplinary priority rankings rather than as discipline-specific preference profiles, population-level estimates, or empirical validation among students. Given the small number of experts in each subgroup, no statistical comparison was conducted between international education, management, and psychology experts. This conservative reporting avoids overinterpreting subgroup differences but also means that disciplinary sensitivity should be examined in future replications.

Criterion-layer weights were ranked as follows: organizational support (0.382) > institutional trust dimension (0.275) > organizational socialization (0.223) > psychological safety (0.120). Organizational support received the highest criterion-level priority weight, followed by institutional trust, organizational socialization, and psychological safety.

**Figure 2:**
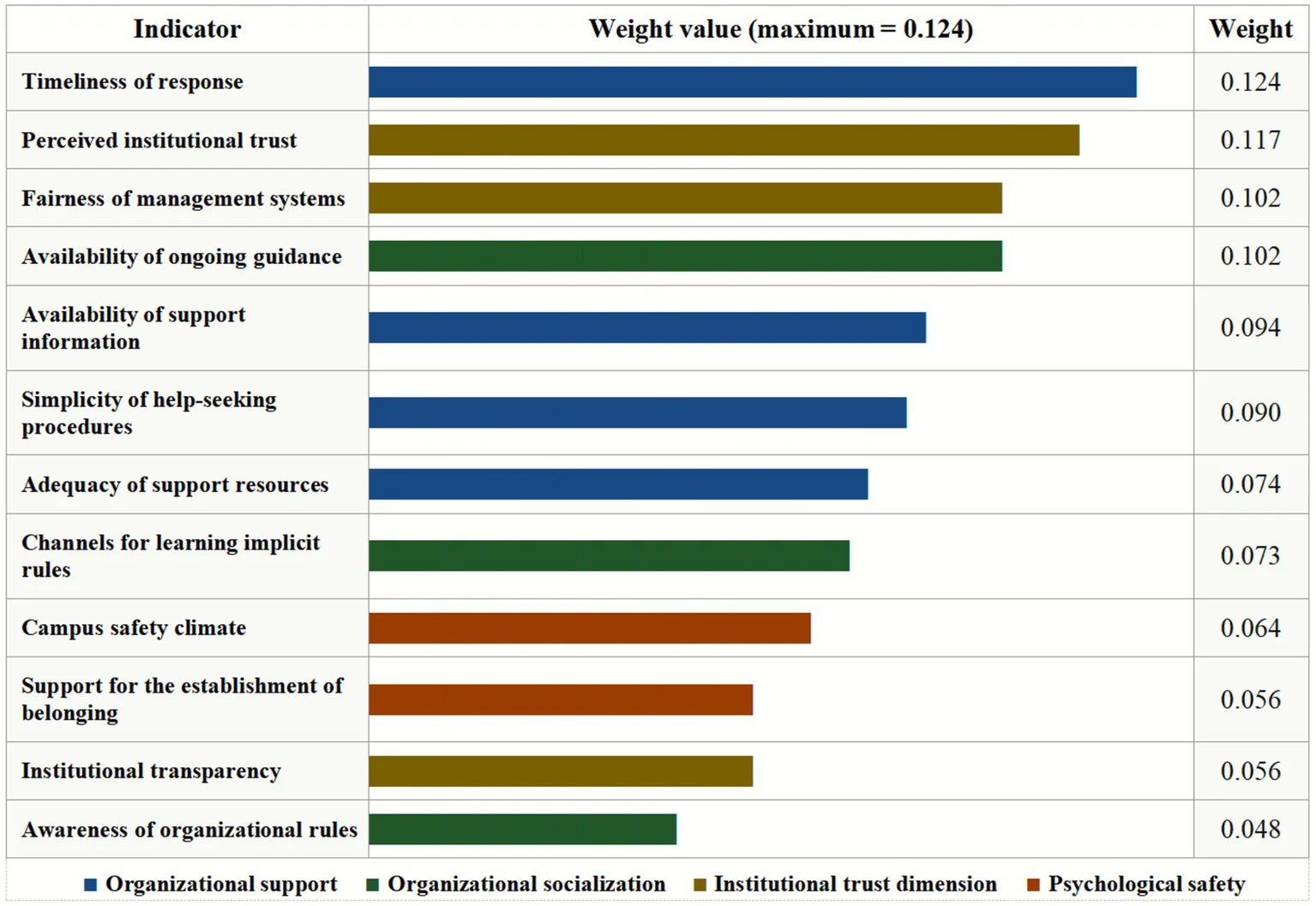
Distribution of weights of key indicators.

Table 5 and Figure 2 show that the top four high-priority indicators were timeliness of response (0.124), perceived institutional trust (0.117), fairness of management systems (0.102), and availability of ongoing guidance (0.102). Timeliness of response ranked first, while adequacy of support resources ranked only seventh (0.074). This should not be interpreted as meaning that resources are unimportant. Rather, the expert ranking suggests that when basic resources exist, their practical value may depend on whether students can recognize, access, and trust them. The ranking therefore indicates that the expert panel prioritized support access, procedural simplicity, and response efficiency above the simple expansion of resource scale.

**Table 5:** Ranking of weights of key AHP indicators.

Rank	Indicator	Criterion layer	Global weight
1	Timeliness of response	Organizational support	0.124
2	Perceived institutional trust	Institutional trust dimension	0.117
3	Fairness of management systems	Institutional trust dimension	0.102
4	Availability of ongoing guidance	Organizational socialization	0.102
5	Availability of support information	Organizational support	0.094
6	Simplicity of help-seeking procedures	Organizational support	0.090
7	Adequacy of support resources	Organizational support	0.074
8	Channels for learning implicit rules	Organizational socialization	0.073
9	Campus safety climate	Psychological safety	0.064
10 (tie)	Support for the establishment of belonging	Psychological safety	0.056
10 (tie)	Institutional transparency	Institutional trust dimension	0.056
12	Awareness of organizational rules	Organizational socialization	0.048

Table reports global weights and relative ranks. These weights represent aggregated expert priorities rather than causal effect sizes. Local indicator weights and the decomposition of global weights are provided in Supplementary Table S11. The complete pairwise-comparison questionnaire and the aggregated criterion-layer judgment matrix are provided in Supplementary Material S8 and Supplementary Table S9, respectively.

Within organizational socialization, availability of ongoing guidance (0.102) received a higher expert priority than channels for learning implicit rules (0.073) and awareness of organizational rules (0.048). This ranking suggests that continuous guidance should be prioritized over one-off rule transmission in support design. Within the institutional trust dimension, perceived institutional trust (0.117) and fairness of management systems (0.102) received higher priority than institutional transparency (0.056), indicating that experts regarded students’ subjective sense of trust and fairness as particularly relevant to psychologically safe support use.

### 4.3 Integrated findings

Integrated findings suggested broad convergence between the qualitative themes and AHP weights. AHP clarified relative expert priorities, while qualitative findings explained why these priorities were meaningful for students’ psychological adaptation experiences. One divergence was that co-national peer networks did not appear as a direct AHP dimension; instead, their effects were reflected indirectly through psychological safety and belonging-related indicators. Table 6 presents the joint display.

**Table 6:** Integrated table of qualitative findings and AHP results.

Qualitative finding	Corresponding AHP indicator	Global weight	Integrated explanation
Support is perceived through visibility, accessibility, and responsiveness rather than resource quantity	Timeliness of response	0.124	These perceptual indicators have higher weights than resource adequacy, suggesting that support should be designed so that students experience it as reachable, responsive, and psychologically usable rather than merely available.
Support is perceived through visibility, accessibility, and responsiveness rather than resource quantity	Availability of support information	0.094	These perceptual indicators have higher weights than resource adequacy, suggesting that support should be designed so that students experience it as reachable, responsive, and psychologically usable rather than merely available.
Support is perceived through visibility, accessibility, and responsiveness rather than resource quantity	Simplicity of help-seeking procedures	0.090	These perceptual indicators have higher weights than resource adequacy, suggesting that support should be designed so that students experience it as reachable, responsive, and psychologically usable rather than merely available.
Support is perceived through visibility, accessibility, and responsiveness rather than resource quantity	Adequacy of support resources	0.074	Resource adequacy remains relevant, but its psychological value may depend on whether resources are recognizable, approachable, and trustworthy enough for students to use.
Organizational socialization is a continuous learning process	Availability of ongoing guidance	0.102	Ongoing guidance is a high-weight indicator, suggesting that experts prioritized continuous guidance over one-off orientation.
Organizational socialization is a continuous learning process	Channels for learning implicit rules	0.073	Informal rule-learning channels support psychological adaptation but are less urgent than sustained guidance.
Institutional mistrust and low psychological safety can make formal support dormant	Perceived institutional trust	0.117	Trust-related indicators have high weights, suggesting that experts viewed institutional trust as a priority condition for psychologically safe support use.
Institutional mistrust and low psychological safety can make formal support dormant	Fairness of management systems	0.102	Fairness helps students judge whether systems are reliable, non-punitive, and safe to approach.
Institutional mistrust and low psychological safety can make formal support dormant	Campus safety climate	0.064	Psychological safety supports students’ willingness to express needs and use formal systems, thereby contributing to psychological adaptation.
Co-national peer networks are both emotional shelter and anxiety amplifier	Campus safety climate	0.064	Co-national peer networks were not entered as an independent AHP dimension. Their relevance is reflected indirectly through psychological safety, belonging, and emotional regulation.
Co-national peer networks are both emotional shelter and anxiety amplifier	Support for the establishment of belonging	0.056	This indirect treatment should be read as a methodological limitation and may underrepresent informal peer processes relevant to psychological adaptation.

The integrated results suggest that formal support becomes psychologically meaningful when students experience it as responsive, continuous, and trustworthy, rather than merely available as a resource. Timeliness of response ranked first (0.124), suggesting that students’ perception of support may be strongly shaped by whether help-seeking produces a quick and clear reply. Perceived institutional trust (0.117) and fairness of management systems (0.102) also ranked highly, supporting the interpretation that students may need to experience formal systems as reliable, confidential, and non-punitive before they are willing to seek help. Availability of ongoing guidance (0.102) further suggests that organizational socialization should be designed as a continuing process rather than concentrated only in orientation, especially in high-uncertainty contexts.

Co-national peer networks require additional qualitative interpretation because their informal effects were only partly captured by AHP weights. Although they were not included as an independent AHP criterion, the interviews suggested that they were relevant to emotional regulation, anxiety transmission, and campus integration. Formal institutional support and informal peer dynamics should therefore be interpreted together, while recognizing that the AHP phase did not empirically estimate the effect of peer networks on adaptation.

## 5 Discussion

### 5.1 Discussion of the core findings

The present study yields four main findings. First, for international students from war-affected regions, organizational support is experienced primarily through visibility, accessibility, and responsiveness rather than through the sheer quantity of available resources. The high ranking of timeliness of response, support information, and procedural simplicity suggests that support must be usable before it can become psychologically meaningful for adaptation. This finding extends perceived organizational support theory by showing that support provision and support perception are not equivalent. In high-vulnerability contexts, formal support matters not merely because it exists, but because students can locate it, approach it without fear, trust it, and experience it as safe enough to use.

Second, organizational socialization appears to operate as an ongoing rather than one-off process. The salience of availability of ongoing guidance suggests that psychological adaptation involves sustained learning of institutional rules, relational pathways, and informal norms. Students need repeated opportunities to understand how the university operates, whom they can approach, and how to interpret formal and informal expectations. For war-affected students, organizational socialization is therefore not only a process of rule learning, but also a reparative process through which they may rebuild predictability and action certainty.

Third, perceived institutional trust appears to function as an activation condition for formal support use. Even when services exist, students may avoid them if they fear stigma, records, or institutional consequences. The high ranking of perceived institutional trust and fairness of management systems indicates that the expert panel regarded reliability, fairness, confidentiality, and non-punitive communication as priority conditions for encouraging psychologically safe help-seeking. This finding is particularly important for students whose prior experiences may have weakened their trust in formal institutions.

Fourth, co-national peer networks serve both protective and risk-amplifying functions. They offer emotional shelter, familiar language, and practical information, but they may also intensify distress through repeated discussion of conflict, family safety, and home-country uncertainty. They may also limit broader campus integration when students remain within closed peer circles. These findings suggest that peer networks should be understood as ambivalent social environments rather than as uniformly positive sources of support.

Taken together, these findings suggest that psychological adaptation among international students from war-affected regions should be understood not only through cross-cultural adjustment but also through support usability, trust in formal systems, psychologically safe help-seeking pathways, and sustained organizational socialization.

### 5.2 Comparison with existing research

This study differs from prior research in several ways. First, it treats the university not as a passive background environment but as a receiving organization that actively shapes psychological adaptation. Existing studies of international student adjustment often emphasize language ability, cultural distance, psychological distress, or social support. These factors remain important, but the present study suggests that formal organizational mechanisms also matter. For students from war-affected regions, the key issue is not simply whether support exists, but whether it can be perceived and used without fear.

Second, the study shifts attention from support quantity to support usability. Prior studies commonly emphasize the availability of social support or institutional resources. The present findings suggest that timeliness of response, support visibility, and procedural simplicity may be more important than resource adequacy. This distinction is theoretically meaningful because it suggests that perceived organizational support should be examined not only in terms of support content but also in terms of accessibility, responsiveness, and psychological safety.

Third, the study identifies perceived institutional trust as a central condition for help-seeking. Existing research has discussed stigma, cultural barriers, and help-seeking reluctance among international students and refugees, but the present study clarifies how institutional trust may link formal systems to actual support use. Students may avoid counseling, academic guidance, or administrative help not because they do not need support, but because they are uncertain about confidentiality, fairness, and possible institutional consequences.

Fourth, the study connects organizational socialization with trauma-informed and forced-migration education perspectives. For war-affected students, socialization is not merely learning how to behave in a new organization. It also involves restoring educational continuity, rebuilding confidence in predictable rules, and developing safe pathways for action. This extends organizational socialization theory into a high-vulnerability educational context in which psychological adaptation is closely tied to institutional predictability and safe help-seeking.

The Chinese university context gives these findings specific meaning. International students are usually managed through schools of international education, academic departments, counselors, supervisors, and administrative units handling student status, scholarships, and visa or residence procedures. For war-affected students, the connection between academic records, administrative procedures, and visa-related services may intensify concern that help-seeking will be documented or misinterpreted. This helps explain why confidentiality, institutional trust, fairness, and response efficiency were especially salient in the findings.

This context also means that institutional trust is not only an abstract attitude toward the university. For international students, academic progress, scholarship status, residence procedures, and access to student services may be perceived as institutionally connected. Even when universities do not use counseling records or support requests in punitive ways, students may still worry that disclosure of distress will be documented, misunderstood, or linked to administrative decisions. This perception helps explain why trust, confidentiality, and non-punitive communication are especially important in the Chinese higher education context.

Alternative explanations and boundary conditions should also be considered. Students’ reluctance to use formal support may not be explained by institutional mistrust alone. Language proficiency, cultural stigma surrounding mental health, lack of familiarity with counseling, time pressure, economic strain, prior administrative experiences, and uncertainty about how Chinese university procedures operate may also reduce help-seeking. Similarly, high priority for response timeliness may reflect experts’ concern with institutional service design rather than students’ own ranking of urgent needs, which could vary by degree level, length of stay, language ability, financial situation, and intensity of conflict-related stress.

The ambivalent role of co-national peer networks also suggests that adaptation processes may differ across students and contexts. Some studies emphasize peer support and friendship as protective for international student adjustment, whereas the present interviews suggest that same-region networks can also transmit anxiety or encourage social closure. This should be interpreted as a boundary condition rather than a contradiction: peer networks may support adaptation when they provide belonging and practical information, but may hinder broader integration when they reinforce worry, isolation, or repeated exposure to distressing home-country news.

### 5.3 Theoretical contributions

This study makes three main theoretical contributions.

First, it extends perceived organizational support theory in a high-vulnerability context by shifting attention from support provision to support usability. In this population, support is theoretically consequential not simply because it exists, but because students experience it as recognizable, approachable, and trustworthy enough to be used. This suggests that future studies of psychological adaptation should distinguish between the formal existence of support and the subjective usability of support.

As introduced in the theoretical framework, support usability can be understood as a three-layer construct: recognizability, approachability, and trustworthiness. The present findings further suggest that these layers are not merely procedural features but psychological conditions that determine whether support is experienced as real, safe, and usable. This three-layer structure clarifies why support provision does not automatically become perceived organizational support.

Second, it conceptualizes perceived institutional trust as an activation condition for formal help-seeking. Rather than functioning as a distant contextual perception, institutional trust may help explain whether students are willing to approach formal university systems when under stress. This finding clarifies a possible psychological process through which trust, fairness, confidentiality, and safety may influence support use.

Third, it advances organizational socialization theory by conceptualizing socialization in this context as continuous reparative socialization. For students whose educational continuity has been disrupted by war-related conditions, socialization involves not only learning rules but also rebuilding predictability, interpretability, and action certainty in a new institutional environment. This makes organizational socialization a psychological adaptation process rather than only an administrative onboarding process (Table 7).

**Table 7:** Summary of theoretical contributions.

Theoretical contribution	Core argument	Difference from existing research
Support usability	Formal support matters not merely because it exists, but because students psychologically experience it as recognizable, approachable, and trustworthy.	Extends perceived organizational support (POS) theory from what organizations provide to how vulnerable students experience and mobilize support for psychological adaptation.
Institutional trust as an activation condition	Trust in formal systems is conceptualized as an activation condition for psychologically safe help-seeking rather than a distant background perception.	Repositions institutional trust as a mechanism that activates or inhibits formal support use.
Continuous reparative socialization	Socialization involves rebuilding predictability, interpretability, and action certainty after educational disruption, thereby supporting psychological adaptation.	Extends organizational socialization theory into high-vulnerability and conflict-sensitive educational contexts where adaptation is also psychological.

### 5.4 Practical implications for psychological adaptation and student-centered support design

The findings have several practical implications for Chinese universities, but these implications concern how institutional arrangements are psychologically experienced by students and how such experiences may support psychological adaptation, rather than management optimization alone. They should be understood as evidence-informed design implications derived from qualitative findings and expert priority rankings; their effectiveness requires future empirical validation through student-level quantitative or intervention studies.

First, universities should design support procedures in ways that allow students to psychologically experience them as recognizable, approachable, and responsive. Since timeliness of response ranked first among all indicators, slow or unclear systems may make existing resources feel functionally unusable. Clear response time expectations, multilingual information platforms, dedicated contact persons, and simplified cross-departmental procedures may help students experience support as recognizable and approachable, thereby strengthening the psychological conditions for help-seeking.

Second, universities should make organizational socialization feel like ongoing guidance rather than one-off orientation. Students from war-affected regions may need repeated explanations of academic expectations, administrative rules, campus norms, and help-seeking channels. Mentor systems, regular check-ins, senior-student peer guidance, and low-threshold consultation channels may help students gradually rebuild predictability and action certainty in the university environment.

Third, universities should make confidentiality and non-punitive intent psychologically credible to students, rather than merely stating them as formal principles. The confidentiality and non-punitive nature of counseling, academic support, and administrative consultation should be communicated clearly and repeatedly. Students should be able to believe that seeking help will not negatively affect academic evaluation, scholarship status, visa renewal, or residence-related procedures. Fair and transparent complaint and feedback mechanisms may further support institutional trust and the experience of psychological safety over time.

Fourth, universities should treat co-national peer networks as part of students’ everyday psychological environment. These networks can provide emotional support and practical information, but they may also amplify anxiety and limit broader integration. Peer mentoring, intercultural exchange, mixed-group activities, and psychological literacy programs may help students support one another without reinforcing distress, thereby supporting psychological adaptation without closing students off from wider campus relationships.

### 5.5 Limitations and future research

This study has several limitations. First, the qualitative sample was modest, purposively and partly snowball-sampled, and drawn from five Chinese universities, which limits the transferability of the findings to other institutional types, regions of China, and national higher education contexts. Second, although broad subregional clusters were reported to improve transparency, the study did not disclose country-level or small-cell demographic information because doing so could increase the risk of deductive identification. The war-affected-region label was used as a contextual sampling category and does not imply uniform individual exposure to conflict, refugee status, or comparable trauma histories. The study also did not further distinguish types of conflict exposure, such as civil war, international conflict, political violence, or direct versus indirect traumatic exposure. Third, the AHP results reflect expert judgments about support-design priority rather than causal effects or empirical quantitative validation among the target student population. The 15-member expert panel was balanced across three professional groups and was appropriate for exploratory structured judgment, but it cannot represent all relevant professional perspectives; subgroup-specific weight comparisons were not reported because each subgroup contained only five experts. Fourth, self-selection bias may exist, as students willing to participate may differ from those who avoided interviews, particularly if the latter had lower institutional trust or greater distress. Fifth, co-national peer networks were not included as an independent AHP dimension. This decision improved managerial actionability but reduced ecological completeness and may underrepresent informal peer processes. Finally, although reflexive and trauma-sensitive procedures were used, researchers’ interpretive assumptions may still have influenced the analysis. The AHP hierarchy treats the four criterion-layer dimensions as analytically separable, although organizational support, institutional trust, socialization, and psychological safety may interact in practice. The resulting weights should therefore be interpreted as decision priorities rather than independent causal contributions; future research may use an analytic network process to examine these interdependencies.

Future research can proceed in several directions. First, larger and more diverse samples across different university types, geographic regions, and conflict backgrounds should be used to examine the transferability of the findings. Future studies should also adopt more fine-grained classifications of conflict type and exposure intensity to examine heterogeneity within war-affected student populations. Second, longitudinal designs could track how organizational support, institutional trust, socialization, and psychological adaptation change over time. Third, future studies could develop and validate quantitative scales for support usability and perceived institutional trust among high-vulnerability international student groups, and then test whether the priority conditions identified here are empirically associated with student-level psychological adaptation. Fourth, informal peer networks should be incorporated more directly into future models as an independent dimension or moderating condition linking institutional support, anxiety transmission, belonging, and psychological adaptation. Fifth, studies using larger expert panels should conduct subgroup sensitivity analyses to compare whether education researchers, management practitioners, and psychology experts assign different priorities to trust, psychological safety, resource adequacy, and procedural accessibility.

## 6 Conclusion

This study examined psychological adaptation among international students from war-affected regions in Chinese universities from a psychological perspective on organizational support and formal help-seeking. Using an exploratory sequential mixed-methods design, it suggested that support usability was central in both participants’ accounts and expert priority rankings. Organizational socialization was interpreted as a continuous reparative process, and perceived institutional trust emerged as a condition that may enable formal help-seeking.

AHP results further indicated that experts prioritized timeliness of response, perceived institutional trust, fairness of management systems, and ongoing guidance over the simple expansion of support resources. Qualitative findings explained why these priorities matter psychologically: unclear procedures, delayed responses, fear of records, and low psychological safety may prevent students from experiencing formal support as usable even when services exist.

Overall, the findings suggest that the central issue is not only whether support resources are formally available, but whether students psychologically experience support systems as recognizable, approachable, trustworthy, and safe to use. Support systems designed around these psychological conditions may create more favorable conditions for psychological adaptation, academic adjustment, and the rebuilding of educational continuity, but their effectiveness should be tested in future empirical studies with larger student samples.

## Data Availability

The original contributions presented in the study are included in the article/Supplementary material, further inquiries can be directed to the corresponding author.
