# Supplementary material for: Support usability, institutional trust, and psychological adaptation among international students from war-affected regions in Chinese universities: a mixed-methods study
Source: Front Psychol. 2026 Aug 6;17:1913815. doi: 10.3389/fpsyg.2026.1913815 (PMC13490183; doi:10.3389/fpsyg.2026.1913815)
Supplement: Supplementary file 1 [file Supplementary_file_1.docx]

**Supplementary Materials**

**Supplementary Table S1. Anonymized Distribution of Student Participants by Region and Educational Level**

| **Region** | **Country/area codes and N** | **Degree levels represented** |
| --- | --- | --- |
| Middle East | M1, M2, M3; N = 12 | Undergraduate / Master / Doctoral |
| North Africa | N1, N2; N = 4 | Undergraduate / Master |
| Sub-Saharan Africa | A1, A2, A3; N = 10 | Undergraduate / Master / Doctoral |
| Other conflict-affected regions | O1, O2; N = 2 | Master / Doctoral |
| Total | All anonymized codes; N = 28 | Undergraduate / Master / Doctoral |

Note: Country/area codes represent distinct anonymized countries or territories. N values refer to student participants, not to the number of countries or areas.

**Supplementary Table S2. Demographic Characteristics of Student Interviewees (N = 28)**

| **Characteristic** | **Category** | **n** | **%** |
| --- | --- | --- | --- |
| Gender | Male | 18 | 64.3 |
|  | Female | 10 | 35.7 |
| Age group | 18–22 years | 5 | 17.9 |
|  | 23–27 years | 14 | 50.0 |
|  | 28 years and above | 9 | 32.1 |
| Degree level | Undergraduate | 8 | 28.6 |
|  | Master’s | 14 | 50.0 |
|  | Doctoral | 6 | 21.4 |
| Length of stay in China | Less than 6 months | 4 | 14.3 |
|  | 6–12 months | 7 | 25.0 |
|  | 1–2 years | 10 | 35.7 |
|  | More than 2 years | 7 | 25.0 |
| Self-rated Chinese communication proficiency | Fluent in communication | 6 | 21.4 |
|  | Basic communication | 15 | 53.6 |
|  | Mainly rely on English | 7 | 25.0 |

**Supplementary Material S3. Semi-Structured Interview Guides**

**Semi-Structured Interview Guide (International Students)**

For internal sampling only, which broad home-country or regional context do you come from? How long have you been studying in a Chinese university? What is your major?

After coming to a Chinese university, what have been the greatest difficulties you have encountered in study and life?

Are you aware of the support measures your university provides for international students (such as academic tutoring, psychological support, or daily-life assistance)? How did you learn about these measures?

When you encountered difficulties, did you seek support from the university? Was the help-seeking process smooth? Was the university’s response timely?

Do you think the university’s support measures have helped your adaptation? In what respects do they need improvement?

Do you interact frequently with students from your own country? What influence do they have on your adaptation?

What do you think of the university’s management systems (such as student status management and visa services)? Do you trust these systems?

What kinds of support measures do you think would help you better adapt to study and life in a Chinese university?

**Semi-Structured Interview Guide (University Staff)**

How long have you been engaged in the management and support of international students? What are your main responsibilities?

How many international students from war-affected regions are there in your university? How is their overall adaptation?

What targeted support measures does the university provide for international students from war-affected regions?

In the process of providing support for these students, what is the greatest difficulty you have encountered?

In your view, what support do these students need most during their adaptation process?

In what respects do you think the university’s support system needs to be optimized?

**Supplementary Table S4. List of Documents Included in the Analysis**

| **Code** | **Document title / type** | **Source (institutional type)** | **Year / Version** |
| --- | --- | --- | --- |
| D01 | General Regulations on International Student Management | Comprehensive key university | 2023 version |
| D02 | Student Handbook of the School of International Education | Language university | 2022 version |
| D03 | Guidelines for International Student Psychological Counseling Services | University of science and engineering | 2024 version |
| D04 | International Student Accommodation Management and Safety Instructions | Finance and economics university | 2023 version |
| D05 | General Student Handbook (University-wide) | Normal university | 2022 version |
| D06 | Internal Procedures for Visa and Residence Permit Application | Comprehensive key university | 2023 revised |
| D07 | Annual Review Measures for International Student Scholarships | Language university | 2023 version |
| D08 | Detailed Rules for Supporting Students with Academic Difficulties | University of science and engineering | 2022 version |
| D09 | Template for Counselor–Student Talk Records (personal information removed) | Finance and economics university | 2024 version |
| D10 | Orientation Syllabus for New International Students | Normal university | 2023 version |
| D11 | Campus Safety and Crisis Intervention Emergency Plan | Comprehensive key university | 2021 version |
| D12 | 2023 Annual Work Report on International Student Affairs | Language university | 2024 |
| D13 | Measures for Disciplinary Actions and Appeals for Students | University of science and engineering | 2023 version |
| D14 | Notices on Optimizing International Student Services (campus intranet) | Finance and economics university | 2024 |
| D15 | Activity Records of Cross-Cultural Adaptation Support Groups (personal information removed) | Normal university | 2024 |

**Supplementary Table S5. Qualitative Themes, Subthemes, and Coding Examples**

| **Core theme** | **Subthemes and typical codes (keywords)** | **Representative student quotation (condensed)** |
| --- | --- | --- |
| The key to perceiving support lies in whether it is visible, accessible, and responsive | Insufficient information visibility (cannot find the entry point, language barriers) | “The university said there was psychological counseling, but I could not find the application procedure.” |
|  | Insufficient channel accessibility (complex procedures, circulation across multiple departments) | “I asked several people and still did not know which department I should go to.” |
|  | Insufficient responsiveness (slow reply, issues left unresolved for a long time) | “After that there was still no clear reply, so I no longer wanted to ask.” |
| Organizational socialization is a continuous process of learning how the university operates | One-off orientation education is insufficient (heard about it but cannot use it) | “A lot was explained during orientation, but when a problem came up, I still did not know what to do.” |
|  | Learning through peers and teachers (relying on senior students, repeated confirmation) | “Only later, after asking senior students, did I gradually learn these procedures.” |
|  | Learning through trial and error (repeated attempts, learning after setbacks) | “I did not understand at the beginning. I only gradually learned after hitting a wall several times.” |
| Co-national peer networks are both emotional shelter and anxiety amplifier | Emotional support and informational companionship (mutual help among people from the same country, relief of loneliness) | “I feel more at ease when I am with friends from my own country.” |
|  | Anxiety amplification (spread of negative news, mutual reinforcement of worry) | “Everyone keeps talking about things back home, which makes me even more anxious.” |
|  | Closed interaction circles (limited social circles, reduced cross-group interaction) | “If I am always with people from my own country, it is harder to truly integrate into the university environment.” |
| Insufficient perceived institutional trust and psychological safety place the support system in a dormant state | Lack of expectation about institutional consequences (fear of being recorded, fear of effects on visa) | “I am afraid that applying for psychological support will be recorded and affect later procedures.” |
|  | Distrust of fairness/confidentiality (fear of disclosure, fear of trouble) | “It is not that there are no resources, but that I am not sure whether using them will cause other problems.” |
|  | Insufficient psychological safety (not daring to speak, bearing it alone) | “The pressure is great, but I do not really dare tell the university proactively.” |

**Supplementary Table S6. Inter-Coder Reliability Details**

| **Indicator** | **Description / Value** |
| --- | --- |
| Number of researchers conducting independent coding | 2 |
| Total number of transcript units coded independently | 196 (paragraph-level meaning units) |
| Number of units agreed upon independently | 177 |
| Number of disagreements resolved through discussion | 19 |
| Random agreement probability (P(e)) | 0.49 (based on category frequencies in the coding framework) |
| Cohen’s Kappa (κ) | 0.81 |
| Disagreement resolution method | Returning to original transcripts, discussing conceptual boundaries until consensus was reached |

**Supplementary Table S7. Profiles of Participating AHP Experts (N = 15)**

| **Expert code** | **Field of expertise** | **Title/Position** | **Years of relevant experience** | **Keywords of expertise** |
| --- | --- | --- | --- | --- |
| E01 | International Education Research | Professor | 18 | International student mobility, cross-cultural adaptation |
| E02 | International Education Research | Associate Professor | 12 | Comparative education, education policy evaluation |
| E03 | International Education Research | Professor | 20 | Higher education internationalization, international student management |
| E04 | International Education Research | Associate Professor | 10 | Educational organizational behavior, AHP application |
| E05 | International Education Research | Assistant Professor | 8 | International student mental health |
| E06 | International Student Management | Deputy Dean, School of International Education | 15 | International student emergency management, funding system |
| E07 | International Student Management | Director, International Student Office | 12 | Visa and student status management, convergence management |
| E08 | International Student Management | Counselor | 10 | Student daily adaptation, crisis intervention |
| E09 | International Student Management | Academic Affairs Secretary | 9 | Course scheduling, academic support processes |
| E10 | International Student Management | Section Chief, International Cooperation Office | 13 | International student policy making, cross-departmental coordination |
| E11 | Psychology | Professor | 22 | Trauma psychology, crisis intervention |
| E12 | Psychology | Associate Professor | 14 | Immigrant mental health, acculturative stress |
| E13 | Psychology | Lecturer/Counselor | 8 | Psychometrics, psychological counseling |
| E14 | Psychology | Professor | 18 | Social psychology, intergroup trust |
| E15 | Psychology | Associate Professor | 11 | Positive psychology, sense of belonging |

**Supplementary Material S8. Complete AHP Expert Rating Questionnaire**

**AHP Expert Rating Questionnaire (Complete Pairwise-Comparison Form)**

Please compare the relative priority of the following factors for optimizing institutional support for the adaptation of international students from war-affected regions. For each pair, first indicate which factor is more important (A, B, or Equal), and then assign an intensity score using Saaty's 1–9 scale: 1 = equal importance; 3 = moderate importance; 5 = strong importance; 7 = very strong importance; 9 = extreme importance; 2, 4, 6, and 8 = intermediate judgments. Reciprocal values are applied automatically when constructing the judgment matrix.

**Part A. Criterion-layer comparisons (6 pairs)**

| **No.** | **Factor A** | **Factor B** | **More important factor (A/B/Equal)** | **Intensity (1–9)** |
| --- | --- | --- | --- | --- |
| 1 | Organizational support | Institutional trust dimension |  |  |
| 2 | Organizational support | Organizational socialization |  |  |
| 3 | Organizational support | Psychological safety |  |  |
| 4 | Institutional trust dimension | Organizational socialization |  |  |
| 5 | Institutional trust dimension | Psychological safety |  |  |
| 6 | Organizational socialization | Psychological safety |  |  |

**Part B. Organizational-support indicator comparisons (6 pairs)**

| **No.** | **Factor A** | **Factor B** | **More important factor (A/B/Equal)** | **Intensity (1–9)** |
| --- | --- | --- | --- | --- |
| 1 | Availability of support information | Simplicity of help-seeking procedures |  |  |
| 2 | Availability of support information | Timeliness of response |  |  |
| 3 | Availability of support information | Adequacy of support resources |  |  |
| 4 | Simplicity of help-seeking procedures | Timeliness of response |  |  |
| 5 | Simplicity of help-seeking procedures | Adequacy of support resources |  |  |
| 6 | Timeliness of response | Adequacy of support resources |  |  |

**Part C. Institutional-trust indicator comparisons (3 pairs)**

| **No.** | **Factor A** | **Factor B** | **More important factor (A/B/Equal)** | **Intensity (1–9)** |
| --- | --- | --- | --- | --- |
| 1 | Perceived institutional trust | Fairness of management systems |  |  |
| 2 | Perceived institutional trust | Institutional transparency |  |  |
| 3 | Fairness of management systems | Institutional transparency |  |  |

**Part D. Organizational-socialization indicator comparisons (3 pairs)**

| **No.** | **Factor A** | **Factor B** | **More important factor (A/B/Equal)** | **Intensity (1–9)** |
| --- | --- | --- | --- | --- |
| 1 | Availability of ongoing guidance | Channels for learning implicit rules |  |  |
| 2 | Availability of ongoing guidance | Awareness of organizational rules |  |  |
| 3 | Channels for learning implicit rules | Awareness of organizational rules |  |  |

**Part E. Psychological-safety indicator comparison (1 pair)**

| **No.** | **Factor A** | **Factor B** | **More important factor (A/B/Equal)** | **Intensity (1–9)** |
| --- | --- | --- | --- | --- |
| 1 | Campus safety climate | Support for the establishment of belonging |  |  |

**Supplementary Table S9. Aggregated Pairwise Comparison Judgment Matrix at the Criterion Layer**

| **Criterion layer** | **Organizational Support** | **Institutional Trust Dimension** | **Organizational Socialization** | **Psychological Safety** | **Local Weight** |
| --- | --- | --- | --- | --- | --- |
| Organizational Support | 1 | 1.658 | 1.203 | 3.763 | 0.382 |
| Institutional Trust Dimension | 0.603 | 1 | 1.613 | 2.087 | 0.275 |
| Organizational Socialization | 0.831 | 0.620 | 1 | 1.601 | 0.223 |
| Psychological Safety | 0.266 | 0.479 | 0.625 | 1 | 0.120 |
| Consistency test | λmax = 4.072 | CI = 0.024 | RI(4) = 0.89 | CR = 0.027 | < 0.10 |

Note: Pairwise comparison entries are rounded to three decimal places for presentation. Criterion-layer weights were calculated from the unrounded aggregated judgment matrix and are reported consistently with the main manuscript.

**Supplementary Material S10. Informed Consent Form**

Title of Study: Support Usability, Institutional Trust, and Psychological Adaptation among International Students from War-Affected Regions in Chinese Universities: A Mixed-Methods Study

**Dear Participant,**

You are invited to participate in an academic study on the adaptation of international students in Chinese universities. Before deciding whether to participate, please read the following information carefully to understand the purpose, procedures, potential risks, and your rights.

1. **Purpose and Background**

This study aims to gain an in-depth understanding of the adaptation experiences of international students from war-affected regions studying at Chinese universities, and to examine how organizational support, management systems, institutional trust, and other factors influence your academic and daily life. The findings will be used to help universities improve support services for international students.

**2. Procedures**
If you agree to participate, you will:

(1) Interview: Take part in an in-depth interview lasting approximately 40–60 minutes. The interview language will be Chinese or English, according to your preference.

(2) Audio recording: The interview will be audio-recorded with your consent for subsequent transcription and analysis. If you prefer not to be recorded, the researcher will take handwritten notes instead.

(3) Member checking: After preliminary analysis, we may contact you briefly to confirm the core meanings you expressed. You will not be asked to revisit any distressing details.

**3. Potential Discomfort and Risks**

(1) Sensitivity: This study adopts a trauma-sensitive interview approach. We do not require you to describe scenes of war violence or painful traumatic memories in detail. The focus is on your campus adaptation experiences and your experience of receiving support.

(2) Emotional fluctuation: If you feel any discomfort or emotional fluctuation during the interview, you may request a pause, skip questions, or withdraw from the interview at any time without any penalty.

(3) Psychological referral: If needed, the researcher can provide you with referral information for the university psychological counseling center.

**4. Privacy and Confidentiality**

(1) Anonymization: All your personal information (name, nationality, specific university name) will be completely removed during transcription. Only anonymized regional codes (e.g., "student from the Middle East region") or pseudonyms will appear in the findings.

(2) Data security: Interview recordings and transcripts will be stored on encrypted hard drives and accessed only by the research team for analysis.

(3) Limits of confidentiality: Except where required by law or with your explicit consent, your personal information will not be disclosed to any third party (including university instructors or administrative staff). Participation in, refusal to participate in, or withdrawal from this study will not affect participants’ visa or residence status, grades, scholarship evaluations, or access to university services.

**5. Voluntary Participation and Right to Withdraw**

Your participation is entirely voluntary. You have the right to decline participation or withdraw unconditionally at any point during the study. Withdrawal will not result in any penalty or loss of benefits.

6. **Consent Signature**

If you have read and understood the above information and agree to participate in this study, please sign below.

·I confirm that I have read and understood this informed consent form.

·I agree to participate in the interview and to audio recording.

·I understand that my participation is voluntary and that my data will be kept confidential.

**Participant's Signature:** __________________________	Date: ________

**Researcher's Signature:** __________________________	Date: ________

**Supplementary Table S11. Decomposition and Reconstruction of the 12 Indicator Weights**

| **Rank** | **Criterion layer** | **Indicator** | **Criterion weight** | **Local weight** | **Global weight** |
| --- | --- | --- | --- | --- | --- |
| 1 | Organizational support | Timeliness of response | 0.382 | 0.3246 | 0.124 |
| 2 | Institutional trust dimension | Perceived institutional trust | 0.275 | 0.4255 | 0.117 |
| 3 | Institutional trust dimension | Fairness of management systems | 0.275 | 0.3709 | 0.102 |
| 4 | Organizational socialization | Availability of ongoing guidance | 0.223 | 0.4574 | 0.102 |
| 5 | Organizational support | Availability of support information | 0.382 | 0.2461 | 0.094 |
| 6 | Organizational support | Simplicity of help-seeking procedures | 0.382 | 0.2356 | 0.090 |
| 7 | Organizational support | Adequacy of support resources | 0.382 | 0.1937 | 0.074 |
| 8 | Organizational socialization | Channels for learning implicit rules | 0.223 | 0.3274 | 0.073 |
| 9 | Psychological safety | Campus safety climate | 0.120 | 0.5333 | 0.064 |
| 10 (tie) | Institutional trust dimension | Institutional transparency | 0.275 | 0.2036 | 0.056 |
| 10 (tie) | Psychological safety | Support for the establishment of belonging | 0.120 | 0.4667 | 0.056 |
| 12 | Organizational socialization | Awareness of organizational rules | 0.223 | 0.2152 | 0.048 |

Note: Local weight = global weight / criterion-layer weight; global weight = criterion-layer weight × local weight. Local weights are reconstructed from the criterion and global weights reported in the main manuscript. Minor differences may occur because displayed values are rounded.

**Supplementary Table S12. Transformation from Qualitative Findings to AHP Indicators**

This table documents how interview and document-analysis findings were transformed into the AHP hierarchy. The AHP indicators were therefore derived from qualitative evidence and used for expert priority ranking, not for causal validation among students.

| **Qualitative core theme** | **Subthemes / coding focus** | **AHP criterion layer** | **AHP indicator(s)** | **Reason for inclusion in AHP** |
| --- | --- | --- | --- | --- |
| Support must be visible, accessible, and responsive to be perceived as real | Information visibility, channel accessibility, response timeliness, perceived resource sufficiency | Organizational support | Availability of support information; simplicity of help-seeking procedures; timeliness of response; adequacy of support resources | These indicators translate students' accounts of functionally usable support into actionable service-design priorities. |
| Organizational socialization is a continuous learning process | One-off orientation is insufficient; learning through teachers and senior students; learning through trial and error | Organizational socialization | Availability of ongoing guidance; channels for learning implicit rules; awareness of organizational rules | These indicators capture how students gradually learn formal rules, implicit norms, and help-seeking pathways. |
| Institutional mistrust and low psychological safety can make formal support dormant | Fear of records, stigma, institutional consequences, unclear confidentiality, and low safety in disclosure | Institutional trust dimension; psychological safety | Perceived institutional trust; fairness of management systems; institutional transparency; campus safety climate | These indicators operationalize trust, fairness, transparency, and safety as conditions that may make formal support more approachable. |
| Co-national peer networks are both emotional shelter and anxiety amplifier | Emotional companionship, familiar language, anxiety transmission, and closed interaction circles | Psychological safety / belonging-related interpretation rather than independent AHP criterion | Support for the establishment of belonging; campus safety climate | Peer networks were retained in the qualitative interpretation but not entered as a separate AHP criterion because the AHP phase prioritized formal institutional design conditions. |

Note. The table clarifies the mixed-methods integration pathway: qualitative themes generated the indicator system, AHP assigned expert priority weights, and the joint display integrated both forms of evidence at the interpretation stage.
